# Clinical characteristics and prognostic nomograms of 12555 non-severe COVID-19 cases with Omicron infection in Shanghai

**DOI:** 10.1186/s12879-023-08582-5

**Published:** 2023-09-16

**Authors:** Chun Yin, Bo Hu, Kunyan Li, Xian Liu, Shuili Wang, Rulin He, Haibing Ding, Mingpeng Jin, Cheng Chen

**Affiliations:** 1 Department of Cardiology, Xinqiao Hospital, Third Military Medical University (Army Medical University), Chongqing, China; 2 Department of Cardiology, the 902Nd Hospital of PLA Joint Service Support Force, Bengbu, China; 3 Department of Radiology, Air Force Hospital of Eastern Theater Command, Malujie Road, Nanjing, China; 4 Department of Pharmaceutical Sciences, Beijing Institute of Radiation Medicine, Beijing, China; 5 Key Laboratory of Arrhythmias of the Ministry of Education of China, Research Center for Translational Medicine, East Hospital, Tongji University School of Medicine, Shanghai, China; 6 The Second Medical Center & National Clinical Research Center for Geriatric Diseases, Chinese PLA General Hospital, Beijing, China

**Keywords:** COVID-19, Omicron, Deterioration, Prognosis, Nomogram

## Abstract

**Background:**

Omicron variant of the severe acute respiratory syndrome coronavirus-2 (SARS-CoV-2) has rapidly become a global threat to public health. Numerous asymptomatic and mild cases had been admitted in shelter hospitals to quickly win the fight against Omicron pandemic in Shanghai. However, little is known about influencing factors for deterioration and length of stay (LOS) in hospitals among these non-severe cases.

**Methods:**

This study included 12,555 non-severe cases with COVID-19 in largest shelter hospital of Shanghai, aiming to explore prognostic factors and build effective models for prediction of LOS.

**Results:**

Data showed that 75.0% of participants were initially asymptomatic. In addition, 94.6% were discharged within 10 days, only 0.3% with deterioration in hospitals. The multivariate analysis indicated that less comorbidities (OR = 1.792, *P* = 0.012) and booster vaccination (OR = 0.255, *P* = 0.015) was associated with the decreased risk of deterioration. Moreover, age (HR = 0.991, *P* < 0.001), number of symptoms (HR = 0.969, *P* = 0.005), time from diagnosis to admission (HR = 1.013, *P* = 0.001) and Cycle threshold (CT) values of N gene (HR = 1.081, *P* < 0.001) were significant factors associated with LOS. Based on these factors, a concise nomogram model for predicting patients discharged within 3 days or more than 10 days was built in the development cohort. In validation cohort, 0.75 and 0.73 of Areas under the curve (AUC) in nomograms, similar with AUC in models of simple machine learning, showed good performance in estimating LOS.

**Conclusion:**

Collectively, this study not only provides important evidence to deeply understand clinical characteristics and risk factors of short-term prognosis in Shanghai Omicron outbreaks, but also offers a concise and effective nomogram model to predict LOS. Our findings will play critical roles in screening high-risk groups, providing advice on duration of quarantine and helping decision-makers with better preparation in outbreak of COVID-19.

**Supplementary Information:**

The online version contains supplementary material available at 10.1186/s12879-023-08582-5.

## Introduction

To date, Corona Virus Disease 2019 (COVID-19), caused by severe acute respiratory syndrome coronavirus-2 (SARS-CoV-2), has globally resulted in more than 0.6 billion confirmed cases and 6 million deaths according to data from WHO. Since February 2022, pandemic of omicron variant has rapidly appeared in Shanghai China. The detection of viral genomes revealed that viruses in this period were classified into BA.2.2 sub-lineage of omicron [1, 2], which is characterized by greater fitness [3], higher infectivity and lower severity for human beings [4, 5]. As of June 1 2022, more than 640 thousand cases, only including 588 deaths, have been identified in Shanghai. Considering the huge population, the number of patients with infection and resultant death should have been higher without Shanghai’s great efforts, including large scale of nucleic acid, quarantine of infected patients in shelter hospitals [1].

Shelter hospitals were implemented for the first time in Wuhan to accommodate for surge of COVID-19 patients in 2020 [6]. Due to advantages of rapid construction, massive scale and low cost [7], shelter hospital had been used to relieve the pressure of health system until the optimal timing to reopen. In shelter hospitals, medical, emotional and social supports were provided to help patients recover and thrive [8, 9]. If patients’ conditions worsen, they could be transferred to higher-level hospitals for advanced treatment [10–12]. When a sudden outbreak occurred in Shanghai, many public buildings were urgently transformed into shelter hospital. Of these, National Convention and Exhibition Centre provided not only 50 thousand beds as the largest hospital, but also large amounts of real-world data from infectious cases at a short time.

Recently, Chinese term for COVID-19 has been renamed from "novel coronavirus pneumonia" to "novel coronavirus infection". China began to manage COVID-19 with measures against Class B infectious diseases, instead of Class A infectious diseases. Authorities have dropped quarantine measures against people infected with novel coronavirus and stopped identifying close contacts or designating high-risk and low-risk areas. COVID-19 cases would receive classified treatment. This major shift of its epidemic response policies might be followed by predictable surge of cases with Omicron infection in the short term. Therefore, analyzing patients’ characteristics and establishing model to predict viral shedding time are essential for screening of cases at high risk and individualized management of massive non-severe cases [13]. Some recent studies revealed the factors which significantly influence severity and mortality of COVID-19 [14–17]. However, few studies gave insights into viral shedding time or length of stay, which was usually treated as secondary outcomes [18]. Although risk factors for viral shedding time were analyzed in previous studies [19, 20], small sample size and information susceptible to recall bias have limited the reliability of results. Further research in large-sample study is needed. Recently, nomogram based on 628 and 197 cases have been tried to integrate characteristics and process massive data in biomedical areas, which makes it available to develop a nomogram model for predicting prognosis [21, 22].

In the present study, real-world data from 12,555 confirmed cases were comprehensively analyzed to reveal clinical feature of patients infected with Omicron, and then to explore risk factors for determination and length of stay in hospital. Moreover, a nomogram was developed and validated to predict the viral shedding time, which provides a basis for the follow-up treatment of inpatients and a reference for efficient management of non-severe Omicron cases.

## Methods

### Study design

A total of 12,555 patients in Shelter Hospital of National Convention and Exhibition Centre were enrolled from April 14 to Aug 30 in 2022. Patients admitted to shelter hospitals met the following criteria: The person was 1) confirmed as positive for COVID-19 by nucleic acid testing, 2) categorized into asymptomatic, mild and moderate groups according to Diagnosis and Treatment Protocol for Novel Coronavirus Pneumonia (Trial Version 9), 3) not complicated with disability of self-care and severe underlying diseases, such as renal failure, heart failure and mental disorders. Children under 18 years, pregnant women and patients refusing to participate in this study or transferred to other hospitals due to personal reason were excluded. Informed consent has been obtained from all the participants. This study was approved by the Ethics Committee and Institutional Review Committee (S2022-769–01).

### Data collection

Basic information, including gender, age, ethnicity, comorbidity, vaccination status, symptoms before their admission, the date of positive RT-PCR test or symptom occurrence for the first time and date of admission and discharge, was recorded when patients were hospitalized. At the same time, whether cases had comorbidities, including cardiovascular disease(e.g.,hypertension), diabetes mellitus, lung disease, hepatic disease, cerebrovascular disease, and kidney disease or who had an immunodeficiency (e.g., human immunodeficiency virus infection, chronic use of corticosteroids, or use of other immunosuppressive drugs), was recorded by questionnaire survey. Patients without any symptom on admission as “initially asymptomatic”. Primary outcomes were deterioration and admission into designated hospitals. The second outcomes were the time from admission to discharge in shelter hospital. If some data from the record were missing or unclear, we re-obtained the data by directly communicating with patients within 24 h. Data were carefully collected from the electronic medical records system and verified by 2 researchers.

RT-qPCR targeting nucleocapsid protein (N) and open reading frame 1ab (ORF lab) gene in the SARS-CoV-2 genome was carried out for patients in shelter hospital every day from admission to discharge. Number of cycles experienced when the fluorescent signal in each reaction tube reaches the set threshold was defined as cycle threshold (CT) value. CT value of less than 35 in shelter hospitals was determined as a positive detection. Detection was completed with Novel Coronavirus nucleic acid test kits (Wuhan EasyDiagnosis Biomedicine) by a qualified laboratory (Shanghai Labway Clinical Laboratory).

### Transfer and discharge criteria

Patients with any of the following conditions would be considered as deterioration and transferred into designated hospitals immediately. 1) Patients’ condition worsened and basic vital signs were unstable. Especially, respiratory rate of patients at rest was more than 30 times per min, or oxygen saturation was less than 93%. 2) Chest imaging showed obvious lesion progression of more than 50%. 3) Serious underlying diseases, including heart failure, respiratory failure or renal failure occurred. 4) Other life-threatening conditions suggested that patients required urgent or specialized treatment.

Patients satisfying all of the following conditions would be discharged from the shelter hospital: 1) CT values of N and ORF lab gene in two consecutive RT-qPCR tests separated by 24 h were both more than 35 2) Temperature returned to normal 3) Relief of symptoms was obtained. Then, length of stay (LOS) in our study was defined as hospitalization duration from admission to discharge in the hospital.

### Clinical prediction models for prediction of stay length in hospital

Branches were randomized assigned to development set or validation set. As a result, the development set consisted of 7,847 patients from 4 branches, which were then defined as A, B, C and D. The validation set consisted of 4,708 patients from 3 branches, defined as E, F and G. The basic information of the training set was fed into a Cox regression model, and a nomogram was plotted for predicting stay length in hospital. Besides, machine learning models, including logistic regression, support vector machine (SVM) and random forest (RF) were used in this study as comparisons. Ten-fold cross-validation was used to select the optimistic hyperparameters in each model.

Finally, data in the validation set were used to test the efficacy of models, with one predicted value for each patient and each model. The performance of prediction was assessed using sensitivity, specificity, accuracy and area under the curve (AUC). Harrell's C-index and calibration curve were used to evaluate the identification and calibration of nomogram. The calibration curve is generated to verify the agreement between the probability predicted by the nomogram and the observed proportions. Decision curve analysis (DCA) was used to evaluate and verify the net benefit rate of nomogram under different risk prediction thresholds.

### Hyperparameter tuning

Ten-fold cross-validation was performed to tune the hyperparameters of the machine learning models. For the LASSO model, each validation procedure was repeated 9 times, with the alpha value changing from 0.1 to 0.9 (step = 0.1). The alpha value that yielded the best result was recorded in each repetition. Ten alpha values that derived from cross-validation procedures were then averaged to obtain the final alpha value, which was found to be 0.5 in this research. Similarly, for the SVM model, the c value was set to 2N, where N ranged from -10 to 10 (step = 0.2). The c value that optimized the result was recorded in each repetition, and the 10 c values were averaged to derive the final c value. In this research, the final c value was determined to be 0.01.

In the RF model, grid search was used in each validation procedure to find the optimized hyperparameters, in which all possible combinations of given parameter were evaluated. In this research, the number of tree changed from 10 to 500 (step = 10), the depth changed from 1 to 20, and the number of splits changed from 1 to 10. The hyperparameters that yielded the best result were recorded. All hyperparameters that derived from cross-validation procedures were then averaged to obtain the final hyperparameter. Finally, the optimized hyperparameters were found to be: number of trees = 100, depth = 8, and number of splits = 5.

### Statistical analysis

All statistical analyses were carried out by using SPSS 22.0 (IBM, White Plains, NY). Fisher’s exact probability test or Chi-Square test was performed to compare difference of proportions for categorical variables. The Mann–Whitney U test or unpaired t-test was performed to analyze the difference between two independent groups of continuous variables. Spearman’s correlation was performed to evaluate correlations between stay length in hospital and other variables. Logistic regression was performed to analyze the independent risk factors of deterioration. Cox regression was employed to calculate hazard ratios of stay length in multi-variable analyses. Whether the COVID-19 patients were deteriorated or discharged was outcome of event. Days of stay in shelter hospital was defined as outcome of time. All *P*-values were two-tailed. 95% confidence intervals (95% CI) were used. *P*-values less than 0.05 were considered statistically significant.

## Results

### Baseline characteristics and outcomes of enrolled patients

**Fig. 1:**
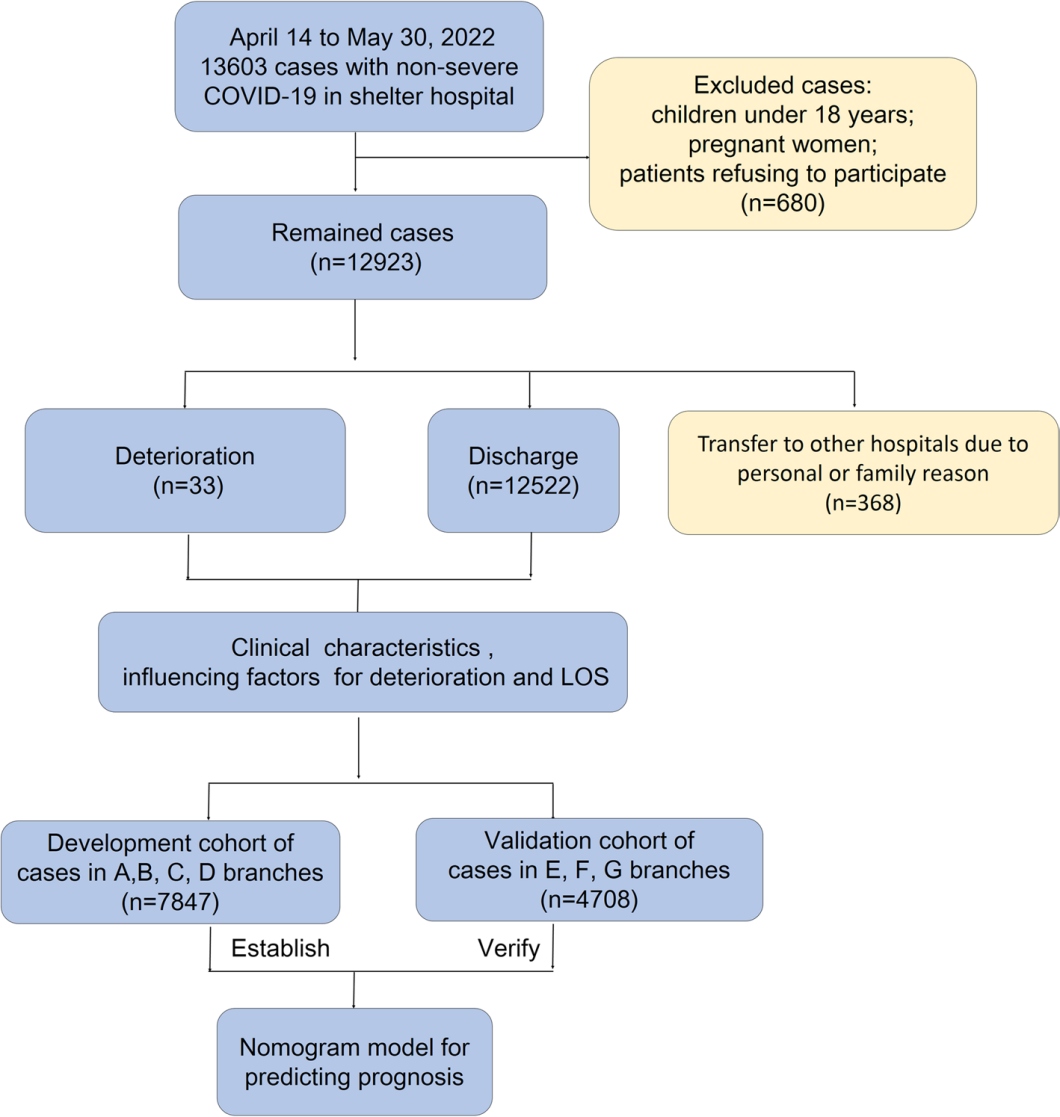
Flow chart of study design

**Fig. 2:**
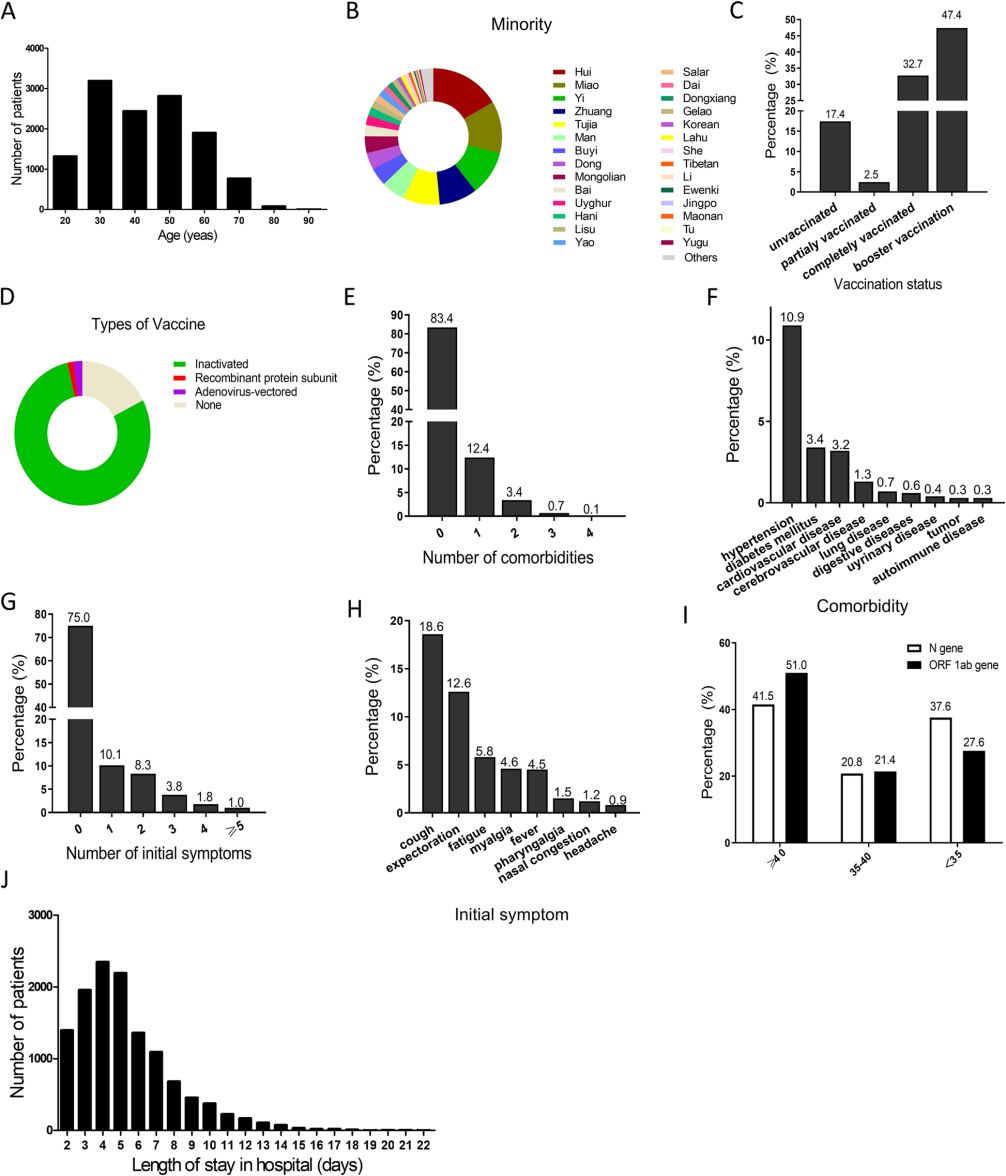
Clinical characteristics and outcomes of non-severe COVID-19 adult patients. **A** Distribution of patients’ age. **B** Proportion of ethnic minority patients. **C** Vaccination status in a total cohort. **D** Types of vaccination that patients have received. **E** Proportion of patients with different numbers of comorbidities. **F** Common comorbidities in all patients. **G** Proportion of patients with different numbers of symptoms. **H** Initial symptoms in all patients. **I** Proportion of patients with different results in nucleic acid test on admission. **J** Distribution of length of stay (LOS) in hospital among patients without deterioration

A total of 12,555 non-severe COVID-19 adult patients in Shelter Hospital of National Convention and Exhibition Centre were included and eventually stratified into development (7847, 62.5%) and validation (4708, 37.5%) cohorts according to hospital branches (Fig. 1). Clinical characteristics and outcomes were described in Table 1 and Fig. 2. In the entire cohort, 8,137 (64.8%) patients were male, and the median age was 41 years. Distribution diagram in Fig. 2A directly revealed that age of adult patients was concentrated in range of 25–55 years old, in which 8,463 patients accounted for 67.4%. Han population was the vast majority (12,254, 97.6%), followed by Hui (50, 0.4%), Miao (37, 0.3%), Yi (32, 0.3%) as shown in Fig. 2B. There were 10,363 patients (82.5%) having received vaccination, including partial (326, 2.6%), complete (4115, 32.8%) and booster (5922, 47.2%) vaccination (Fig. 2C). Inactivated vaccine was administered in most participants (9921, 79.0%) (Fig. 2D). Only 2,080 (16.6%) patients had comorbidities, and 1553 patients (12.4%) with 1 comorbidity (Fig. 2E). The most common comorbidity was hypertension (1363, 10.9%), followed by diabetes (432, 3.4%), cardiovascular disease (397, 3.2%) and cerebrovascular disease (161, 1.3%) (Fig. 2F).

**Table 1:** Baseline characteristics of enrolled patients

	**Total patients** **(*n* = 12 555)**	**Development Cohort** **(*n* = 7 847)**	**Validation Cohort** **(*n* = 4 708)**
Gender			
Male	8137 (64.8%)	4814 (61.3%)	3323 (70.6%)
Female	4418 (35.2%)	3033 (38.7%)	1385 (29.4%)
Age (years)	41 (31–53)	41 (31–53)	41 (30–53)
Race/ethnicity			
Han	12,254 (97.6%)	7653 (97.5%)	4601 (97.7%)
Others	301 (2.4%)	194 (2.5%)	107 (2.3%)
Marital Status			
Married	8273 (65.9%)	5250 (66.9%)	3023 (64.2%)
Unmarried	4282 (34.1%)	2597 (33.1%)	1685 (35.8%)
Comorbidity			
With comorbidities	2080 (16.6%)	1259 (16.0%)	821 (17.4%)
Without comorbidities	10,475 (83.4%)	6588 (84.0%)	3887 (82.6%)
Contact with overseas personnel			
No	12,516 (99.7%)	7831 (99.8%)	4685 (99.5%)
Yes	39 (0.3%)	16 (0.2%)	23 (0.5%)
Vaccination status			
Unvaccinated	2192 (17.5%)	1371 (17.5%)	821 (17.4%)
Vaccinated	10,363 (82.5%)	6476 (82.5%)	3887 (82.6%)
Clinical symptom			
Asymptomatic	9355 (74.5%)	5862 (74.7%)	3493 (74.2%)
Symptomatic	3200 (25.5%)	1985 (25.3%)	1215 (25.8%)
Location of definite diagnosis			
In community	5983 (47.7%)	3785 (48.2%)	2198 (46.7%)
At checkpoints of nucleic acid detection	1934 (15.4%)	1205 (15.4%)	729 (15.5%)
At fever clinic	2434 (19.4%)	1515 (19.3%)	919 (19.5%)
In study or work unit	2198 (17.5%)	1342 (17.1%)	856 (18.2%)
Time from diagnosis to admission (days)	3 (2–6)	3 (2–6)	3 (2–5)
Results of RT-PCR on the day of admission			
Positive	4733 (37.7%)	3140 (40.0%)	1593 (33.8%)
Negative	7822 (62.3%)	4707 (60.0%)	3115 (66.2%)
Disease deterioration			
No	12,522 (99.7%)	7822 (99.7%)	4700(99.8%)
Yes	33 (0.3%)	25 (0.3%)	8 (0.2%)
Length of hospital stay (days)	5 (3–7)	5 (3–7)	5 (4–7)

Values are n (%) or median (interquartile range)

*Abbreviations*: *RT-PCR* reverse transcription polymerase chain reaction

In addition to baseline characteristics, initial symptom, results of RT-PCR and outcomes were also comprehensively analyzed. 75.0% of participants were initially diagnosed with asymptomatic infection, and 10.1% with only one symptom (Fig. 2G). Cough was the most common symptom before hospital admission (18.6%), followed by expectoration (12.6%), fatigue (5.8%) and myalgia (4.6%) (Fig. 2H). All the participants received SARS-CoV-2 PCR tests on admission. Surprisingly, the proportion of participants with ≥ 40 CT values of N gene and ORF 1ab gene were 41.5% and 50%, respectively (Fig. 2I). As shown in Figure S1A and B, CT values of ORF 1ab gene and N gene were closely related (Spearman r = 0.953, *P* < 0.001), however CT values of N gene were lower than those of ORF 1ab gene ( 37.7 vs. 40, *P* < 0.001). Then, dynamic changes of viral load were analyzed. Four different patterns, represented by 4 patients of No.5128, 5924, 13,125 and 13,648, were shown in Figure S1C. Eventually, 33 participants (0.3%) were judged to have deteriorated in shelter hospital, and then transferred to designated hospitals for further treatment (Table 1). Among remained participants, the median stay length in hospital was 5 days (interquartile range: 3–7 days). Distribution curve in Fig. 2J demonstrated that most of participants (94.6%) were discharged within 10 days, 26.7% discharged within 3 days. At early stage of Shanghai Omicron outbreak, many cases can not be immediately sent to shelter hospitals as soon as they were confirmed as positive for COVID-19 by nucleic acid testing. The time from first diagnosis to admission had been added in Figure S5 A. Therefore, viral shedding times actually consisted of two parts: time from first diagnosis and length of stay in shelter hospitals. As showed in Figure S7 B, the median duration of disease was 9 days (interquartile range: 7–12 days). Distribution curve demonstrated that most of participants (99.1%) were recovered within 21 days, 64.2% recovered within 10 days. These results suggested that it was difficult to predict prognosis only based on RT-PCR test on admission.

### Risk and protective factors for deterioration in the hospital

**Fig. 3:**
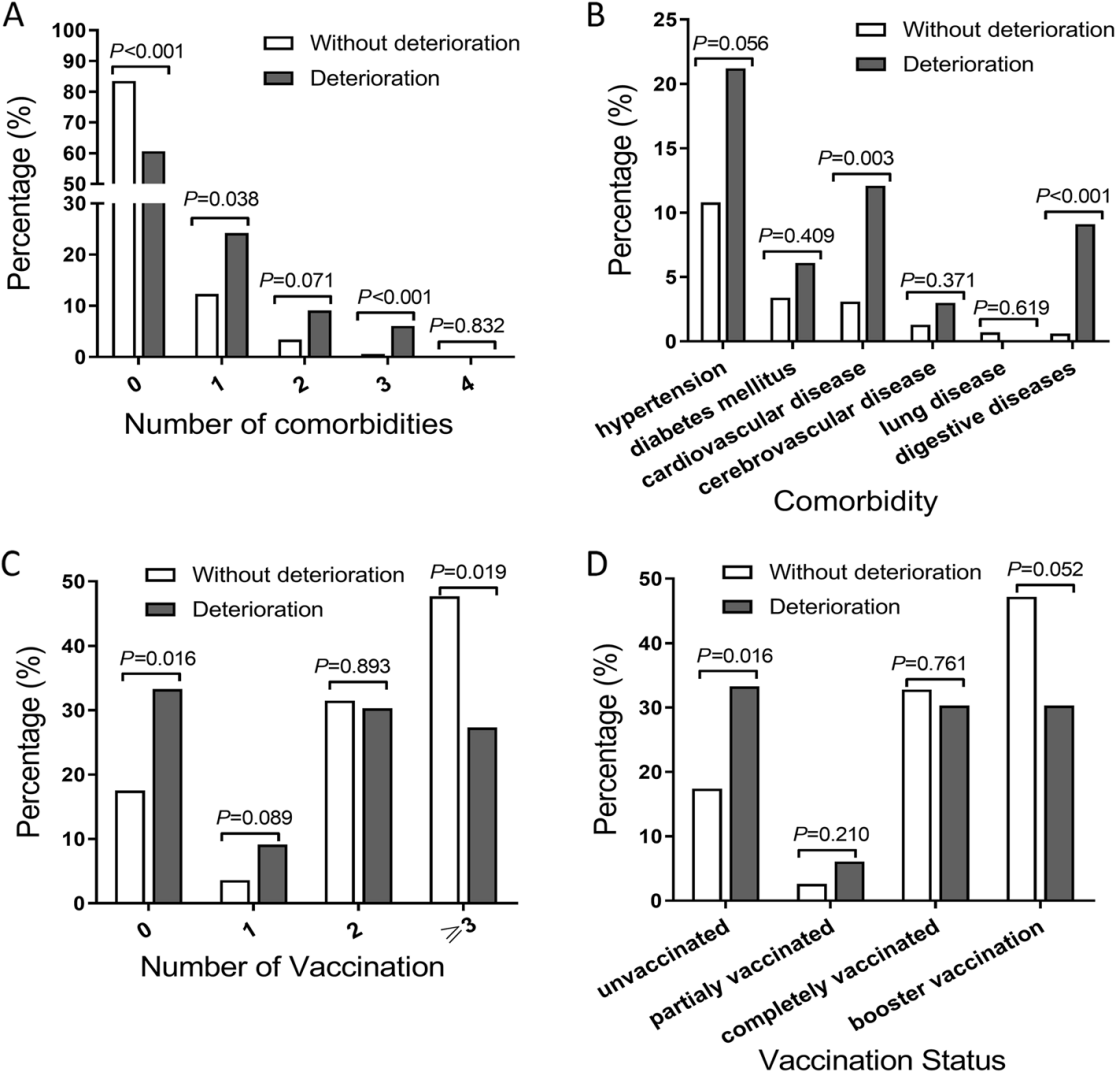
Comorbidities and vaccination as influencing factors for deterioration. Number (**A**) and types (**B**) of comorbidities in deterioration group and non- deterioration group subjects. Number (**C**) and status (**D**) of vaccination in deterioration group and non- deterioration group

Then, risk factors for deterioration were explored with univariate and multivariate analysis of real-world data. In Table S1, results of univariate analysis demonstrated that there were no significant differences in gender, ethnicity, initial symptom and results of RT-PCR on admission between patients with or without deterioration. However, patients who deteriorated in shelter hospital were older (*P* = 0.022), more likely to have comorbidity (*P* < 0.001) and more likely to be unvaccinated (*P* = 0.016). As shown in Fig. 3A, percentage of patients with 1 or 3 comorbidities in deterioration group was significantly higher than that in non- deterioration group (24.2% vs. 12.4% *P* = 0.038; 6.1% vs. 0.7% *P* < 0.001, respectively). Further, results in Fig. 3B revealed that percentage of patients with cardiovascular disease or digestive disease was markedly elevated in deterioration group (12.1% vs.3.2% *P* = 0.003; 9.1% vs. 0.6% *P* < 0.001, respectively.). In contrary, percentage of patients with at least 3 vaccinations in deterioration group was significantly less than that in non- deterioration group (27.3% vs. 47.7% *P* = 0.019, Fig. 3C). Similarly, percentage of patients with booster vaccination tended to be less in deterioration group, though difference was not significant (30.3% vs. 47.2% *P* = 0.052, Fig. 3D). Furthermore, age, comorbidity and vaccination status, which had statistical difference in univariate analysis of Table S1, were included in multivariate logistic regression analysis. Multivariable logistic regression analysis (Table 2) demonstrated that the number of comorbidities was independently associated with the increased risk of deterioration (OR = 1.792, 95% CI: 1.137–2.824, *P* = 0.012). Compared with patients without vaccination, those with booster vaccination were less likely to have deterioration in shelter hospital (OR = 0.255, 95% CI: 0.085–0.769, *P* = 0.015). Taken together, number of comorbidities was an independent risk factor, while booster vaccination an independent protective factor. Then, we attempted to build logistic regression, SVM and RF models to predict the occurrence of deterioration. However, the efficacy of all models were unsatisfactory due to the small sample size in deterioration group (AUC = 0.49, 0.56 and 0.51, respectively, Figure S2).

**Table 2:** Risk factors of deterioration with multivariate logistic regression analysis in total patients

Variables	OR (95% CI)	*P* value
Age	1.024 (0.994–1.055)	0.114
**Number of comorbidity**	**1.792 (1.137–2.824)**	0.012*
Vaccination status		
Unvaccinated	1.000	
Partially vaccinated	1.862 (0.397–8.741)	0.431
Completely vaccinated	0.602 (0.230–1.577)	0.301
** Booster vaccination**	**0.255 (0.085–0.769)**	0.015*

Age, comorbidity and vaccination status, which had statistical difference in univariate analysis of Table S1, were included in multivariate logistic regression analysis. Logistic regression was performed for all cases (*n* = 12,555). * means *P* < 0.05

*Abbreviations*: *OR* odds ratio, *CI* confidence interval

### Influencing factors for LOS of non-severe cases with Omicron infection

**Fig. 4:**
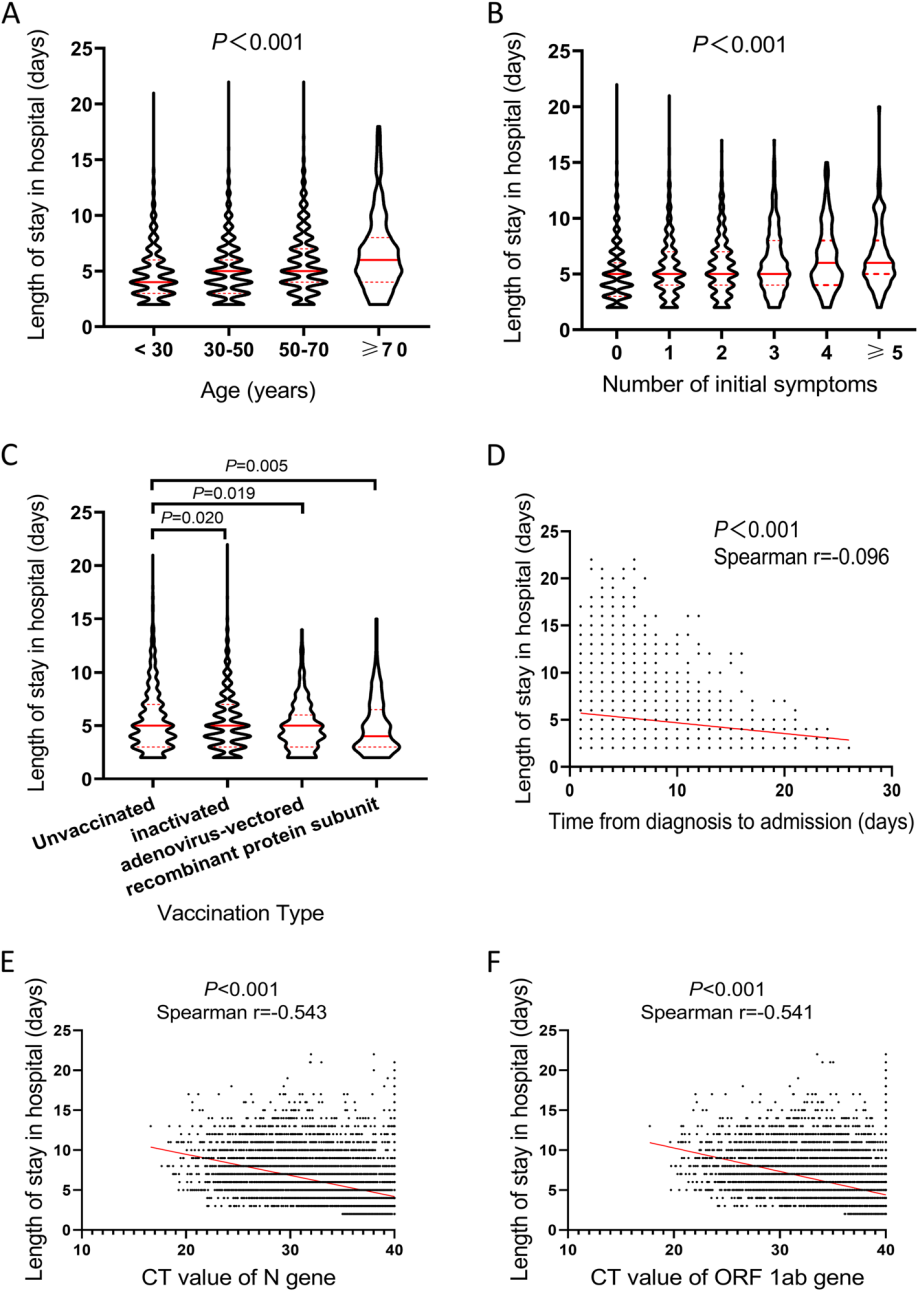
Influencing factors for LOS in hospital. **A** LOS of patients with different ages. **B** LOS of patients with different numbers of symptoms. **C** LOS of patients with different types of vaccination. **D** Correlation analysis between LOS and time from diagnosis to admission. **E** Correlation analysis between LOS and CT values of N gene. **F** Correlation analysis between LOS and CT values of ORF 1ab gene. Red lines represent fitted curves

Then, influencing factors for length of stay (LOS) in shelter hospital were analyzed. Results of univariate analysis showed that LOS was significantly associated with ethnicity, marital status, comorbidity, vaccination status, initial symptom and results of RT-PCR on admission(all *P* < 0.05, Table S2). In addition, patients with older age or more symptoms had longer LOS in shelter hospital (both *P* < 0.001,Fig. 4A and B). Compared with unvaccinated patients, those with inactivated, adenovirus-vectored or recombinant protein subunit vaccines significantly had shorter LOS (*P* = 0.020, *P* = 0.019, *P* = 0.005, respectively) (Fig. 4C). In Fig. 4D, an inversely proportional relationship was observed between LOS in shelter hospital and time from diagnosis to admission (Spearman r = -0.096, *P* < 0.001). Similarly, LOS in shelter hospital was also negatively correlated with CT values of N gene (Spearman r = -0.543, *P* < 0.001, Fig. 4E) or ORF 1ab gene (Spearman r = -0.541, *P* < 0.001, Fig. 4F). Then, the characteristics including age, number of comorbidity, number of symptom, vaccination status, time from diagnosis to admission and CT values of N gene on admission, which had statistical difference or correlation in univariate analysis of Table S2 and Fig. 3, were included in time-dependent Cox regression analysis. As demonstrated in the Figure S6, the rank of time was significantly associated with partial residuals for age, number of comorbidity, number of symptom, time from diagnosis to admission and CT values of N gene (all *P* values < 0.05). Besides, the survival curves for both groups were crossed at multiple locations. These results suggested that time-Dependent Cox Regression Model could be more suitable than traditional Cox regression model. As shown in Table S3, age, number of comorbidity and number of initial symptoms were associated with longer LOS. While, time from diagnosis to admission and CT values of N gene were correlated with shorter LOS (all *P* value < 0.001). We have revised this point in Results of our manuscript.

### Establishment and verification of model for predicting viral shedding time

**Fig. 5:**
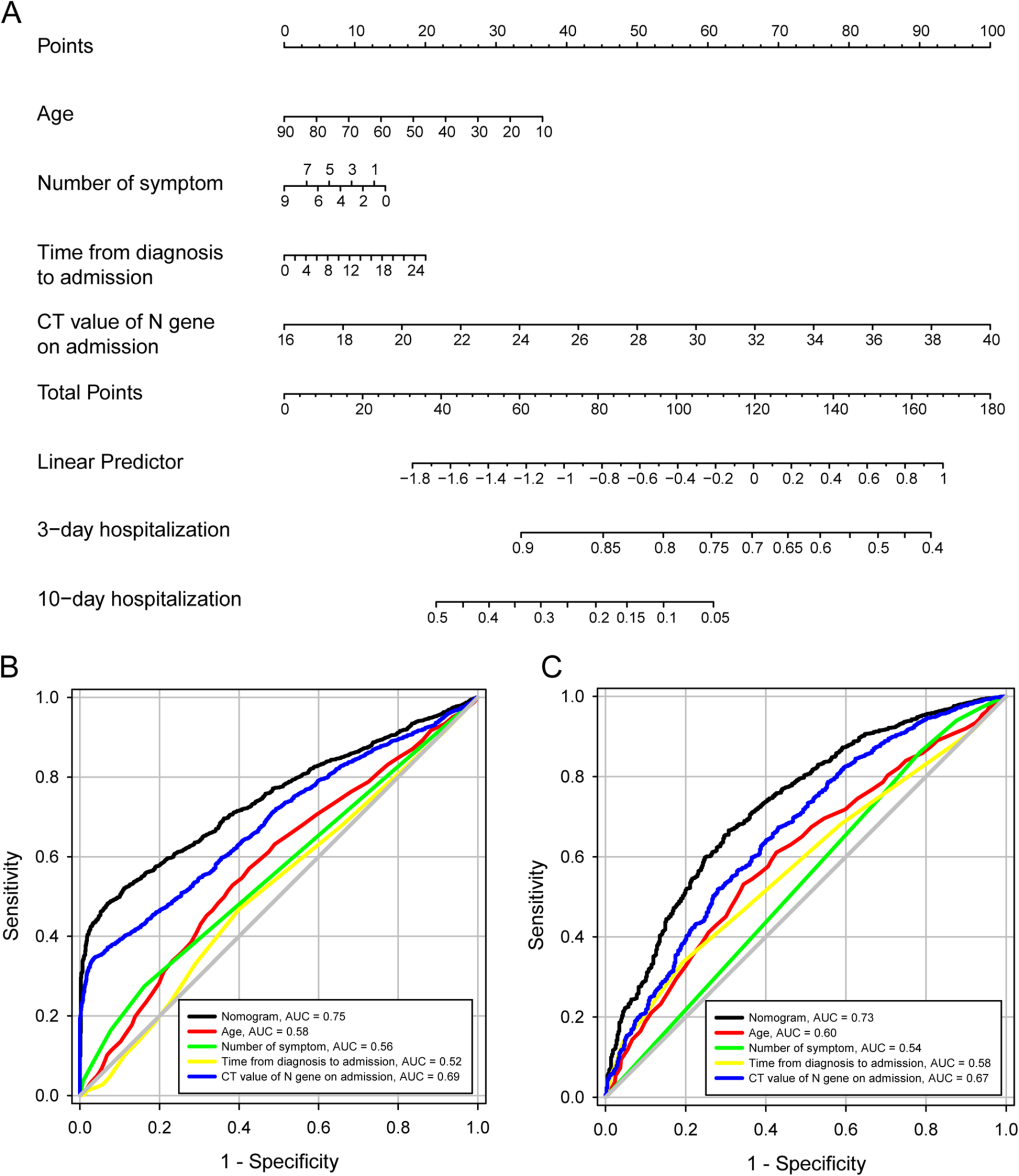
The nomogram based on Cox regression model and the comparison of performance with single univariable. **A** The nomogram derived from cox regression model. **B** The comparison between nomogram and single univariable for predicting 3-day hospitalization. **C** The comparison between nomogram and single univariable for predicting 10-day hospitalization

**Fig. 6:**
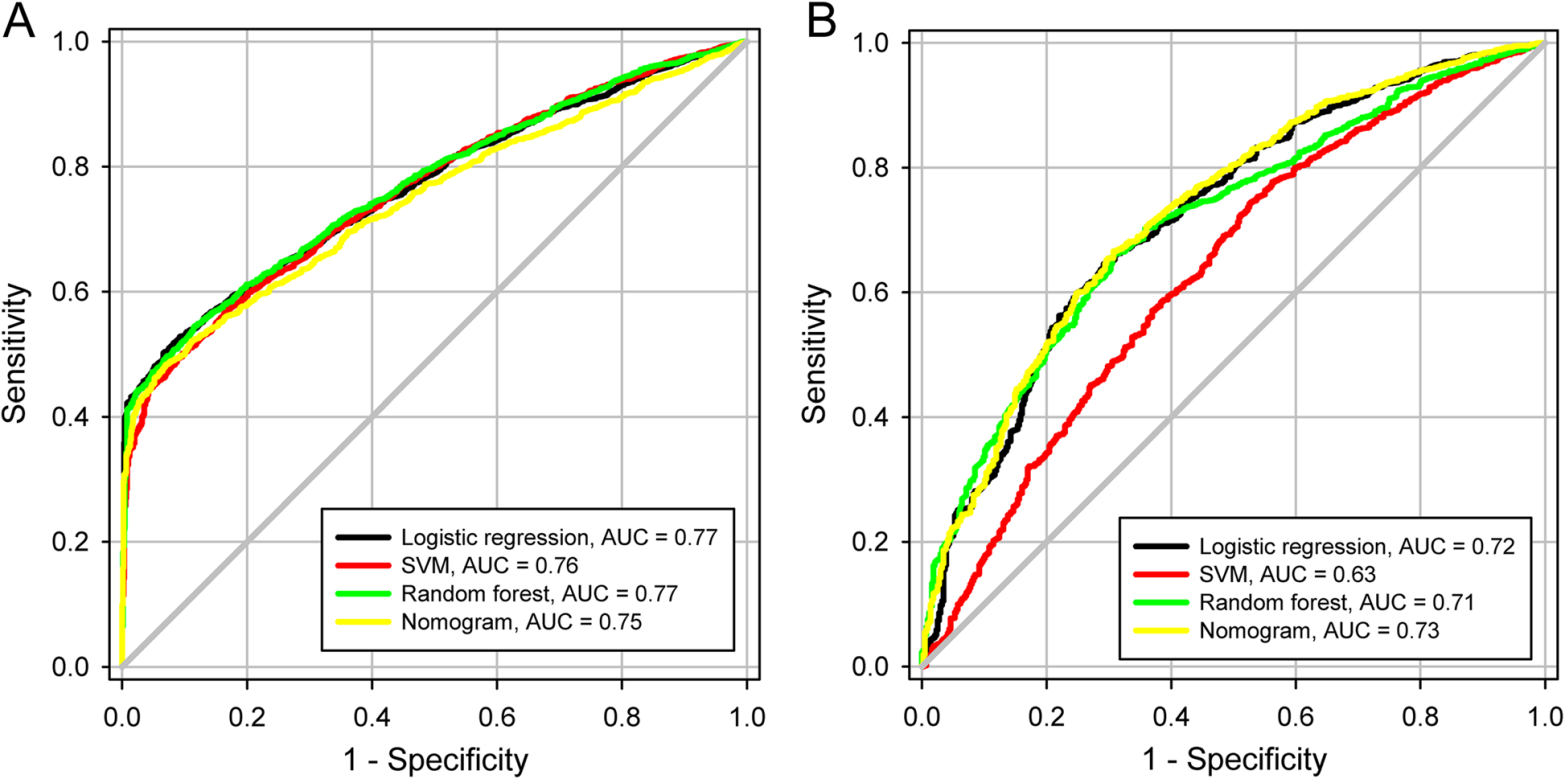
The ROC curve of all models for prediction of length of hospitalization. **A** Prediction for hospitalization within 3 days. All models had comparable predictive performance. **B** Prediction for hospitalization over 10 days

To effectively manage patients and plan for medical resource allocation, a nomogram (Fig. 5A) based on Cox regression model was trained to predict patients discharged within 3 days or more than 10 days. The nomogram combined with multivariate variables (Fig. 5B) was superior to each single univariate in predicting patients discharged within 3 days (AUC = 0.75), including age, number of symptoms, time from diagnosis to admission, and N gene CT value at admission (AUC = 0.58, 0.56, 0.52, and 0.69 separately). For the prediction of patients discharged more than 10 days (Fig. 5C), the nomogram (AUC = 0.73) also superior to each single univariate (AUC = 0.60, 0.54, 0.58, and 0.67 separately). The C-index of 0.721 ± 0.007 and the calibration curve showed good agreement between the predicted and observed probability of 3-day and 10-day hospitalization. The DCA of training set was consistent with validation set (Figure S3), indicating the nomogram had good efficacy and replicability. In Fig. 6A and B, the logistic regression model, SVM and RF showed comparable predictive performance for both 3-days (AUC = 0.77, 0.76, and 0.77, separately) and 10-day (AUC = 0.72, 0.63, and 0.71, separately) hospitalization compared with the nomogram.

## Discussion

China has adopted many important measures to effectively control Omicron epidemics in Shanghai [23]. In this study, real-world data of 12, 555 COVID-19 cases in Shelter Hospital of National Convention and Exhibition Centre were comprehensively analyzed to provide a novel insight for understanding clinical characteristics of non-severe COVID-19 patients and risk factors of their short-term prognosis. Furthermore, a concise and effective nomogram model to predict the length of hospital stay was established and verified for the first time. Although patients would no longer be quarantined in the shelter hospitals nowadays, the asymptomatic or mild patients are still encouraged to stay home and avoid infecting others. Our research on prediction of length of hospital stay may still be useful in advising the public on the duration of self-quarantine at home in order to reduce the risk of transmission of infection without excessive home quarantine. It could serve as a useful reference for professionals of public health and policymakers of healthcare to manage infected cases better and adjust pandemic control strategies.

In this study, 75.0% individuals in shelter hospital were initially asymptomatic, with 94.6% discharging within 10 days. In addition, only 0.3% individuals were transferred to designated hospitals due to deterioration. Similarly, previous studies in Wuhan and southern California also demonstrated that Omicron tended to cause milder symptoms, shorter hospital duration and better outcomes [24–27]. In addition, the severe cases had been excluded from shelter hospitals on admission [19, 28], which further contributed to low rate of deterioration. However, results of univariate and multivariate analysis suggested that patients who were older, unvaccinated, or with comorbidity should be treated more cautiously due to the higher risk of deterioration in shelter hospital. Consistent with previous reports [29], we also found that patients with hypertension and coronary disease tended to get worse prognosis. These findings may be explained by possibly acute cardiac injury suffering from affected angiotensin-converting enzyme 2 (ACE2) signaling pathways and uncontrolled release of pro-inflammatory cytokines [30, 31]. This emphasized the importance to protect patients with these chronic diseases [32]. Notably, vaccination, especially booster vaccination, can effectively protect patients from deterioration. In China, three types of vaccines, including inactivated, adenovirus-vectored and recombinant protein subunit vaccine were widely used to prevent COVID-19. This finding was supported by a previous study, which found that death, admission to ICU and invasive mechanical ventilation were all observed in unvaccinated patients except for one death case. However, in this study, partially vaccination was simply defined as completion of 1or 2 doses of COVID-19 vaccine, and full vaccination as completion of 3 doses of vaccine [28]. We carefully classified the population according to the type, dose and company of vaccines. For the first time, it is confirmed that booster vaccination was an independent protective factors for deterioration during Omicron waves. Moreover, we found that 3 or more doses of vaccine remarkably reduced rates of deterioration, which implied that boosting with heterologous or homologous vaccines both improved protective effect [33, 34]. Taken together, these findings suggests that accelerating coverage of vaccination, especially among the elderly, might be an effective strategy to fight against COVID-19 pandemic. It has been shown that all models for predicting deterioration of 0.3% were unsatisfactory due to small sample size and limited characteristics. Therefore, the predictive model would be optimized in larger cohort with a long-term follow-up visit.

Another highlight in this study is development and validation of a concise and effective nomogram for predicting length of hospital stay, which is critically important for optimized planning of resource allocation and efficient management of shelter hospital. Considering that most patients in shelter hospital were initially asymptomatic, length of hospital stay approximately corresponds to viral shedding time [35]. Data in this study showed a median length of hospital stay of 5 days, as compared to about 16 days for those infected with alpha strain in Wuhan Fangcang shelter hospitals [13, 36]. Most patients discharged within 10 days, which was consistent with dynamic change of viral load in other studies regarding Omicron variants [37]. Our results showed that CT values of N and ORF 1ab gene on admission, semi-quantitative proxies of viral load, were closely related with LOS. Similarly, previous studies have demonstrated that CT value is associated with biochemical and hematological markers [38, 39], disease severity [40, 41] and mortality [42] in COVID-19 patients. Unexpected, CT value of N gene was lower than ORF 1ab gene in the same patient. One explanation could be that transcriptomic level of N gene in both of genomic and subgenomic RNA is significantly more than ORF1ab gene only in genomic RNA [43]. In addition, a recent study found that, compared to N region, there were more mutations in ORF1ab region [2]. Mutations in primer-binding region might lead to false-negative results. Accordingly, CT values of N gene seems more sensitive and suitable for viral load assessment and prognosis prediction. Besides, patient's age, pre-hospital time and initial symptoms reasonably affected the recovery time. Consistently, a previous study has revealed a similar trend that adults gradually recover slower from the infections as they aged [28]. Ultimately, pre-hospital time and initial symptoms were included in a predictive model of LOS for the first time, though they were semi-subjective values susceptible to recall bias. A recent study has clarified that clinical symptoms of patients with mild Omicron infection were more complex and needed to be differentiated from influenza [44]. Moreover, results showed that nomogram could be used to approximately select patients discharging within 3 days or more than 10 days, with similar efficacy to machine learning models. This concise model could provide a novel visual interface to aid in management of bed demand and communication with patients in shelter hospitals.

A recent study including 165,760 COVID-19 cases in Shanghai has also reported several factors that influence LOS in shelter hospitals [35]. It provides valuable guidance for better understanding characteristics in patients with mild or asymptomatic Omicron infection. Some results are similar with those in our study. 90% of patients were discharged on day 10, consistent with 94.6% of patients in our study. Besides, both of studies found that the LOS for adult patients was positively correlated with age. These confirmed the reliability of our findings. However, only univariate analysis was performed in the previous study. COX regression analysis was used to conduct multivariate analysis of LOS in our study. Number of initial symptoms, time from diagnosis to admission, CT values of the N gene were found to be independent factors. In addition, the influencing factors for determination have not attracted enough attention before. Recent change of epidemic response policies in China might lead to huge number of cases with Omicron infection, so identifying high-risk groups is the key point of epidemic control. We found that number of comorbidities and vaccination type is closely related to determination. Moreover, a concise and effective nomogram model to predict the virus shedding time was established and verified for the first time, which has unique significance in clinical practice.

This study had a few inevitable limitations. First, bias might be potentially introduced in the retrospective cohort study because of some self-reported characteristics, such as symptoms. Second, we did not have access to history of smoking, result of CT scan and laboratory tests for all patients. Third, lack of external validation limited wider application of the results in this single-centered study.

## Conclusion

Collectively, this study not only provides important evidence to deeply understand clinical characteristics and risk factors of short-term prognosis in Shanghai Omicron outbreaks, but also offers a concise and effective nomogram model to predict LOS. It is believed that our findings will play critical roles in screening high-risk groups, providing advice on duration of quarantine and helping decision-makers with better preparation in outbreak of COVID-19.

### Supplementary Information

**Additional file 1:** **Figure S1.** Results of RT-qPCR targeting nucleocapsid protein (N) and open reading frame 1ab (ORF lab) gene. **Figure S2.** The ROC curve of logistic regression, SVM and RF models to predict the occurrence of deterioration in hospital. **Figure S3.** The test of fit of nomogram. **Figure S4.** Length of stay in shelter hospital for patients with deterioration. **Figure S5.** Treatment of traditional Chinese medicine in shelter hospital. **Figure S6.** Proportional hazards assumption and Kaplan-Meier curve for Cox analysis. **Figure S7.** Distribution curve of time from first diagnosis to admission (A) and duration of disease (B). **Table S1.** Characteristics of severe and non-severe patients. **Table S2.** Risk factors for length of stay in hospital calculated with univariable analysis in development cohort. **Table S3.** Risk factors of LOS with Time-Dependent Cox Regression analysis in development cohort.

## Data Availability

The datasets used and/or analysed during the current study are available from the corresponding author on reasonable request.
