# Supplementary material for: Clinical characteristics and prognostic nomograms of 12555 non-severe COVID-19 cases with Omicron infection in Shanghai
Source: BMC Infect Dis. 2023 Sep 16;23:606. doi: 10.1186/s12879-023-08582-5 (PMC10504722; doi:10.1186/s12879-023-08582-5)
Supplement: Supplementary file 1 — Additional file 1: Figure S1. Results of RT-qPCR targeting nucleocapsid protein (N) and open reading frame 1ab (ORF lab) gene. Figure S2. The ROC curve of logistic regression, SVM and RF models to predict the occurrence of deterioration in hospital. Figure S3. The test of fit of nomogram. Figure S4. Length of stay in shelter hospital for patients with deterioration. Figure S5. Treatment of traditional Chinese medicine in shelter hospital. Figure S6. Proportional hazards assumption and Kaplan-Meier curve for Cox analysis. Figure S7. Distribution curve of time from first diagnosis to admission (A) and duration of disease (B). Table S1. Characteristics of severe and non-severe patients. Table S2. Risk factors for length of stay in hospital calculated with univariable analysis in development cohort. Table S3. Risk factors of LOS with Time-Dependent Cox Regression analysis in development cohort. [file 12879_2023_8582_MOESM1_ESM.doc]

**Figure S1.** Results of RT-qPCR targeting nucleocapsid protein (N) and open reading frame 1ab (ORF lab) gene. (A) Correlation analysis between CT values of N gene and ORF 1ab gene in the same patients. (B) Comparison of CT values for N gene and ORF 1ab gene in the same patients. (C) Dynamic changes of CT values for N gene and ORF 1ab gene in viral load, represented by 4 patients of No.5128, 5924, 13125 and 13648.


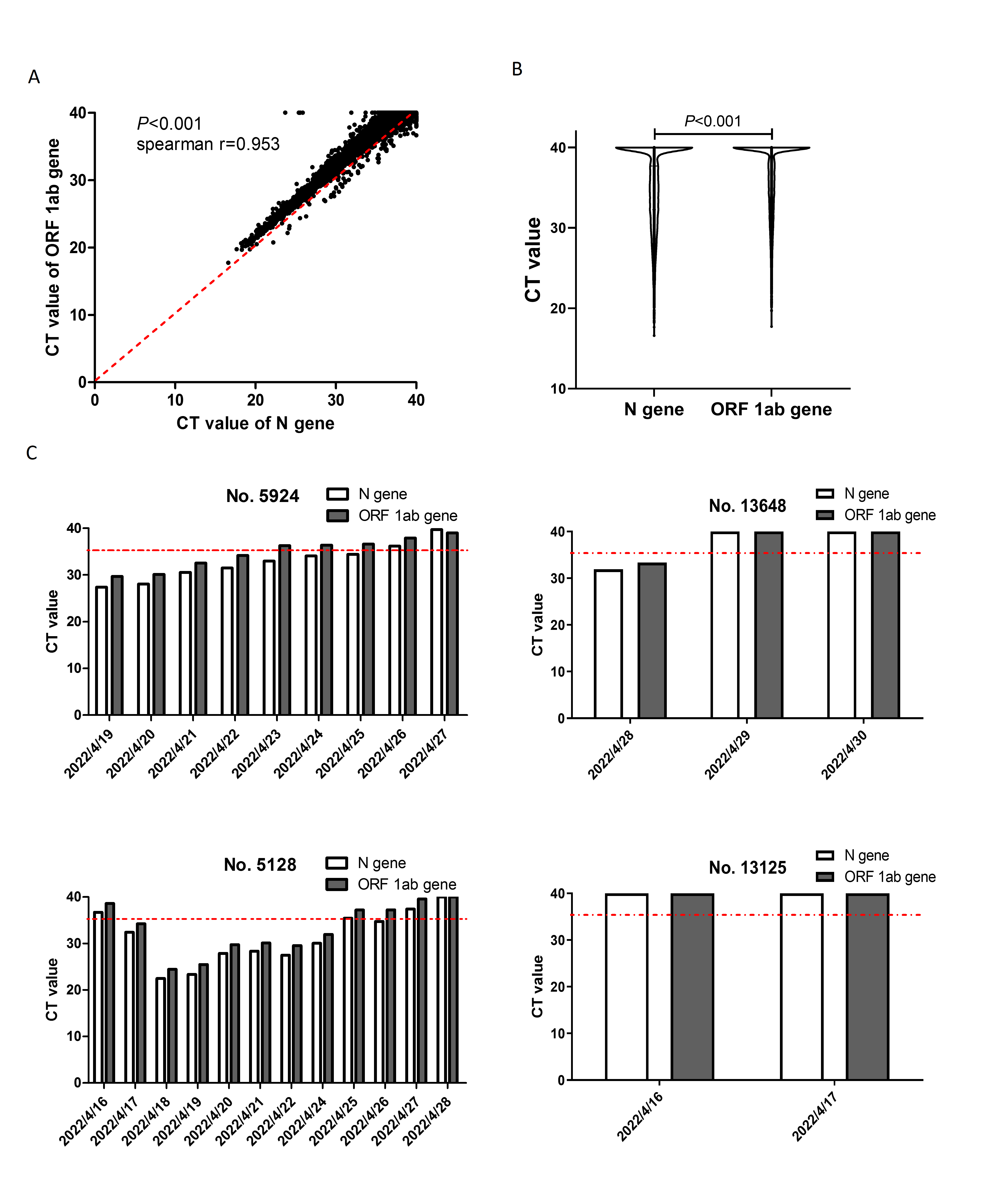


**Figure S2.** The ROC curve of logistic regression, SVM and RF models to predict the occurrence of deterioration in hospital.


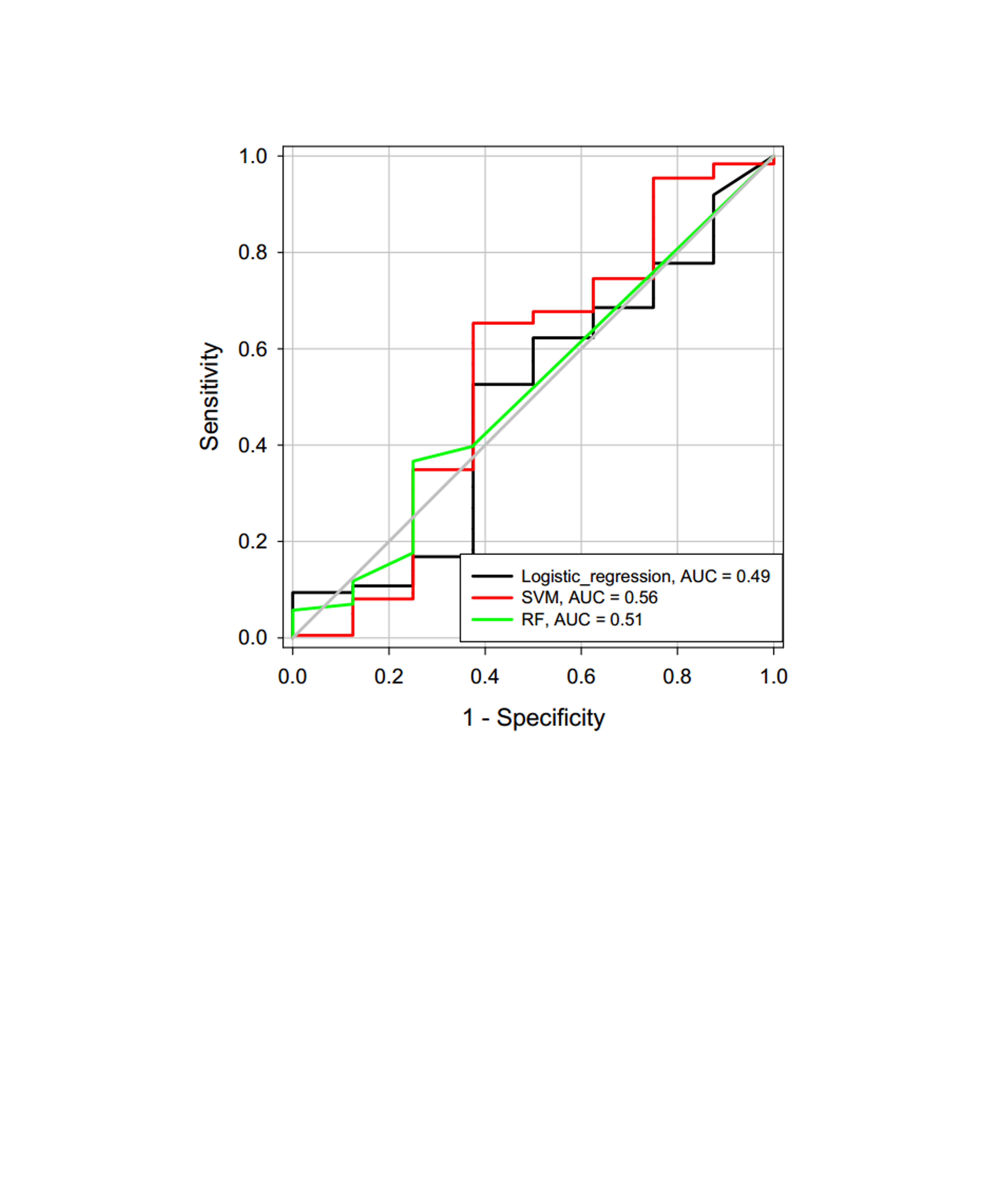


**Figure S3.** The test of fit of nomogram. (A) The calibration curve showed good agreement between the predicted and observed probability of 3-day and 10-day hospitalization. (B) The DCA of training set was consistent with validation set, indicating the nomogram had good efficacy and replicability.


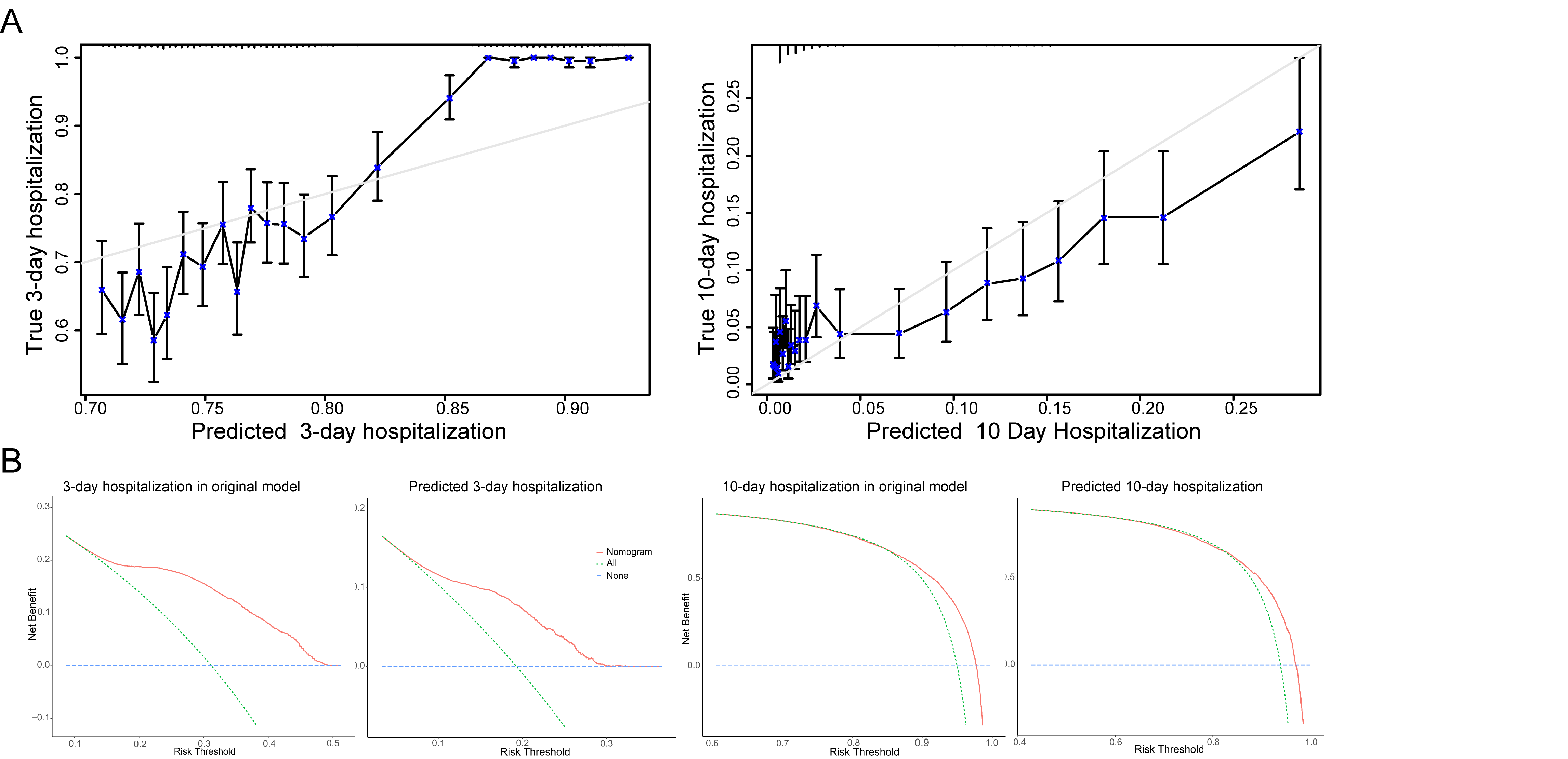


**Figure S4.** Length of stay in shelter hospital for patients with deterioration.


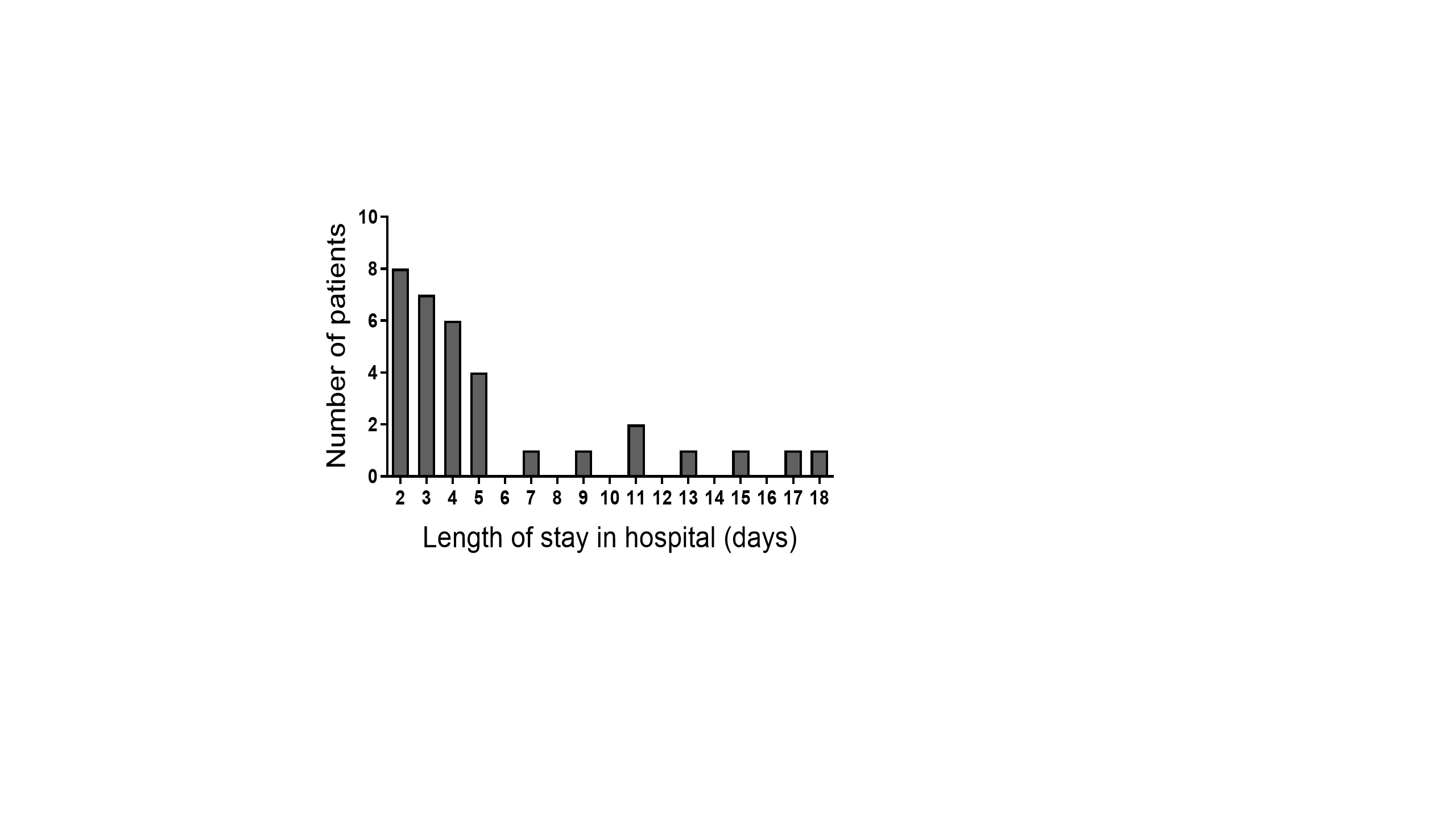


**Figure S5.** Treatment of traditional Chinese medicine in shelter hospital.Proportion of patients taken with different numbers and kinds of TCM (A, B). Proportion of patients taken with TCM in deterioration group and non- deterioration group(C).


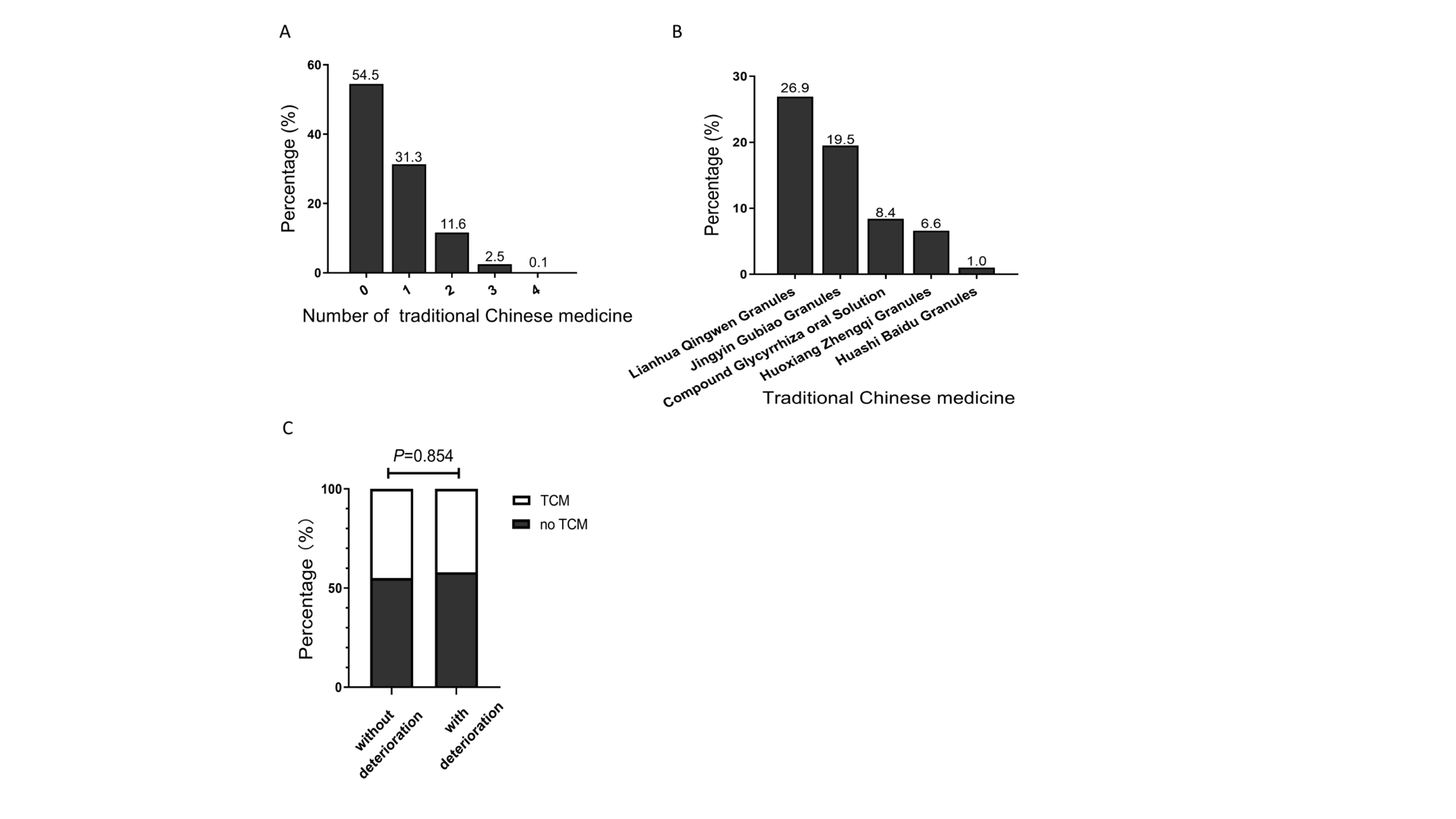


**Figure S6.** Proportional hazards assumption and Kaplan-Meier curve for Cox analysis. Correlation between the rank of time and partial residuals for age, number of comorbidity, number of symptom, time from diagnosis to admission or CT values of N gene.(A-E) Kaplan-Meier curve of vaccination status.(F)

**
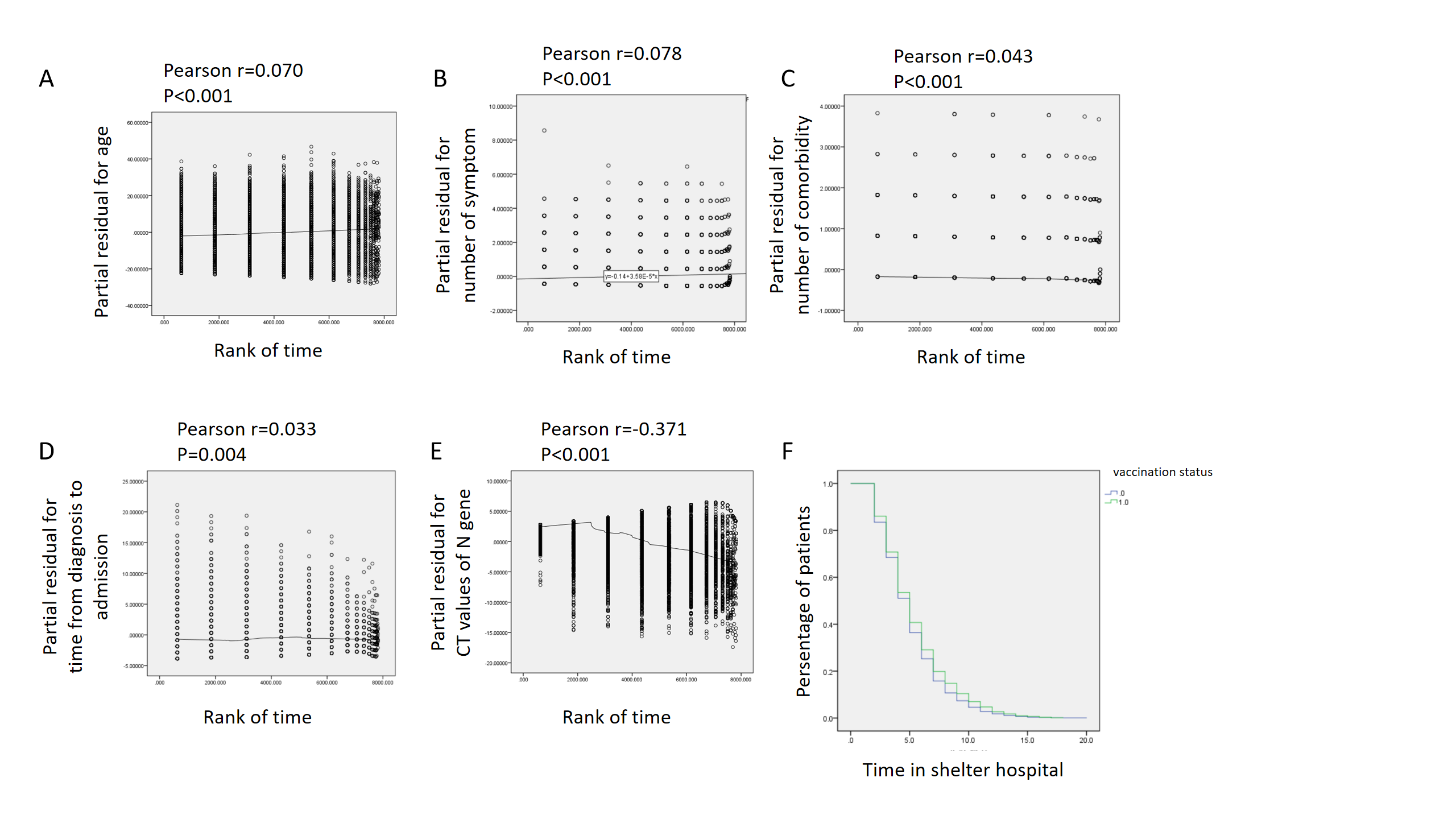
**

**Figure S7.** Distribution curve of time from first diagnosis to admission (A) and duration of disease (B).


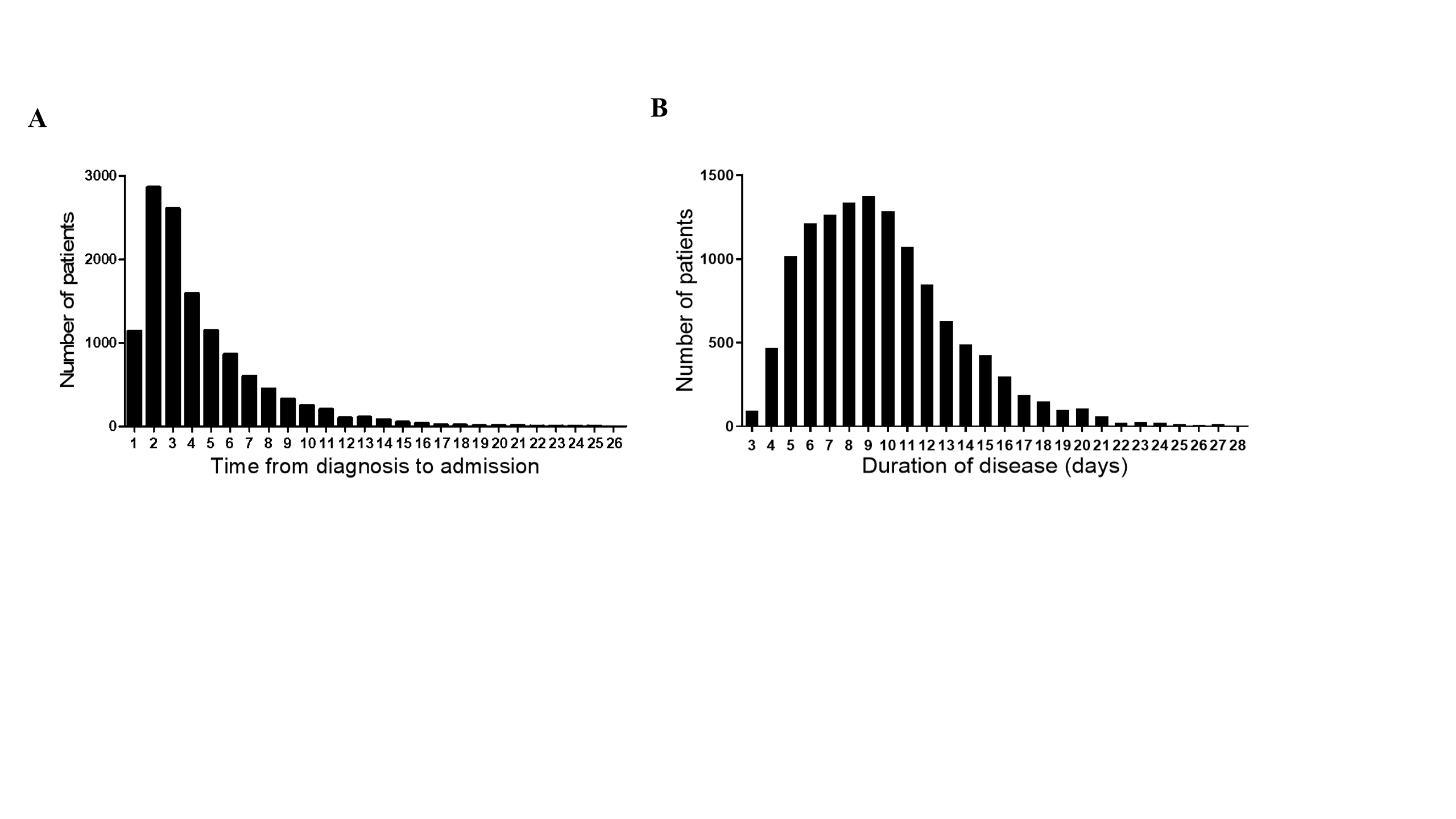


**Table S1. Characteristics of severe and non-severe patients**

|  | **Total**  **(n=12 555)** | **Severe**  **(n=33)** | **Non-severe**  **(n= 12522)** | ***P* value** |
| --- | --- | --- | --- | --- |
| **Gender** |  |  |  |  |
| Male | 8137 (64.8%) | 19 (57.6%) | 8118 (64.8%) | 0.384 |
| Female | 4418 (35.2%) | 14 (42.4%) | 4404 (35.2%) |  |
| **Age (years)** | 41 (31-53) | 53 (30-67) | 41 (31-53) | **0.022^*^** |
| **Race/ethnicity** |  |  |  |  |
| Han | 12254 (97.6%) | 33 (100%) | 12221 (97.6%) | 0.367 |
| Others | 301 (2.4%) | 0 (0%) | 301 (2.4%) |  |
| **Marital Status** |  |  |  |  |
| Married | 8273 (65.9%) | 23 (69.7%) | 8250 (65.9%) | 0.645 |
| Unmarried | 4282 (34.1%) | 10 (30.3%) | 4272 (34.1%) |  |
| **Comorbidity** |  |  |  |  |
| With comorbidities | 2080 (16.6%) | 13 (39.4%) | 2067 (16.6%) | **＜0.001^*^** |
| Without comorbidities | 10475 (83.4%) | 20 (60.6%) | 10455 (83.4%) |  |
| **Contact with overseas personnel** |  |  |  |  |
| No | 12516 (99.7%) | 33 (100%) | 12483 (99.7%) | 0.748 |
| Yes | 39 (0.3%) | 0 (0%) | 39 (0.3%) |  |
| **Vaccination status** |  |  |  |  |
| unvaccinated | 2192 (17.5%) | 11 (33.3%) | 2181 (17.5%) | **0.016^*^** |
| vaccinated | 10363 (82.5%) | 22 (66.7%) | 10341 (82.5%) |  |
| **Clinical symptom** |  |  |  |  |
| asymptomatic | 9355 (74.5%) | 27 (81.8%) | 9328 (74.5%) | 0.335 |
| symptomatic | 3200 (25.5%) | 6 (18.2%) | 3194 (25.5%) |  |
| **Location of definite diagnosis** |  |  |  |  |
| In community | 5983 (47.7%) | 17 (51.5%) | 5966 (47.7%) | 0.629 |
| At checkpoints of  nucleic acid detection | 1934 (15.4%) | 3 (9.0%) | 1931 (15.4%) |  |
| At fever clinic | 2434 (19.4%) | 9 (27.3%) | 2425 (19.4%) |  |
| In study or work unit | 2198 (17.5%) | 4 (12.2%) | 2194 (17.5%) |  |
| **Time** **from diagnosis to admission (days)** | 3 (2-6) | 3 (2-7) | 3 (2-6) | 0.990 |
| **Results of RT-PCR on the day of admission** |  |  |  |  |
| Positive | 4733 (37.7%) | 15 (45.5%) | 4718 (37.7%) | 0.357 |
| Negative | 7822 (62.3%) | 18 (54.4%) | 7804 (62.3%) |  |
| CT value of N gene | 37.7 (32.3-40.0) | 37.9 (29.7-40.0) | 37.7 (32.3-40.0) | 0.512 |
| CT value of ORF 1ab gene | 40.0 (34.4-40.0) | 40.0 (31.4-40.0) | 40.0 (34.4-40.0) | 0.598 |

Abbreviations:RT-PCR=reverse transcription polymerase chain reaction. CT= cycle threshold.nucleocapsid protein (N). open reading frame 1ab (ORF lab) gene

Note: Severe group (n=33): patients were judged to have deteriorated in shelter hospital, and then transferred to ICU or designated hospitals for further treatment; Non-severe group (n=12522):patients were discharged without deterioration in shelter hospital.

Values are n (%) or median (interquartile range).

*P* value was calculated by Fisher's exact test or Mann-Whitney U test. **^*^** means *P*<0.05.

**Table S2. Risk factors for length of stay in hospital** **calculated with univariable analysis in development cohort**

|  | **Length of stay in hospital (days)** | ***P* value** |
| --- | --- | --- |
| **Gender** |  |  |
| Male | 5 (3-7) | 0.065 |
| Female | 5 (3-7) |
| **Comorbidity** |  |  |
| With comorbidities | 5 (4-7) | **＜0.001^*^** |
| Without comorbidities | 5 (3-7) |
| **Contact with overseas personnel** |  |  |
| No | 5 (3-7) | 0.217 |
| Yes | 5 (3-5) |
| **Vaccination status** |  |  |
| unvaccinated | 5 (4-7) | **0.010^*^** |
| vaccinated | 5 (3-7) |
| **Clinical symptom** |  |  |
| asymptomatic | 5 (3-6) | **＜0.001^*^** |
| symptomatic | 5 (4-7) |
| **Results of RT-PCR on the day of admission** |  |  |
| Positive | 6 (5-8) | **＜0.001^*^** |
| Negative | 4 (3-5) |

Abbreviations: CT= cycle threshold. N gene= nucleocapsid protein gene.

P value was calculated by Mann-Whitney U test. * means P<0.05.

**Table S3. Risk factors of LOS with Time-Dependent Cox Regression analysis in development cohort.**

| Variables | HR | *P* value |
| --- | --- | --- |
| **Age** | **Exp(-0.025+0.011×ln(t))** | **<0.001** |
| **Number of symptom** | **Exp(-0.284+0.158×ln(t))** | **<0.001** |
| Vaccination status | Exp(-0.095+0.045×ln(t)) | 0.305 |
| **Number of comorbidity** | **Exp(-0.266+0.175×ln(t))** | **<0.001** |
| **Time from diagnosis to admission** | **Exp(0.063-0.037×ln(t))** | **<0.001** |
| **CT values of N gene on admission** | **Exp(0.373-0.176×ln(t))** | **<0.001** |

Abbreviations: RT-PCR=reverse transcription polymerase chain reaction. HR=hazard ratio. CT= cycle threshold. N gene= nucleocapsid protein gene.

Age, number of comorbidity, number of symptom, vaccination status, time from diagnosis to admission and CT values of N gene on admission, which had statistical difference or correlation in univariate analysis of Table S2 and Figure 3, were included in time-dependent Cox regression analysis. Regression was performed for cases in development cohort (n=7,847). * means P<0.05.
